# A combined MR and CT study for precise quantitative analysis of the avian brain

**DOI:** 10.1038/srep16002

**Published:** 2015-10-30

**Authors:** Daniel Jirak, Jiri Janacek, Benjamin P. Kear

**Affiliations:** 1 MR Unit, Department of Diagnostic and Interventional Radiology, Institute for Clinical and Experimental Medicine, Vídeňská 1958/9, 140 21 Prague, Czech Republic; 2 Institute of Biophysics and Informatics, 1st Medicine Faculty, Charles University, Prague, Czech Republic; 3 Department of Biomathematics, Institute of Physiology, The Czech Academy of Sciences, Vídeňská 1083, 142 20 Prague, Czech Republic; 4 Museum of Evolution, Uppsala University, Norbyvägen 16, SE-752 36 Uppsala, Sweden; 5 Palaeobiology Programme, Department of Earth Sciences, Uppsala University, Villavägen 16, SE-752 36 Uppsala, Sweden

## Abstract

Brain size is widely used as a measure of behavioural complexity and sensory-locomotive capacity in avians but has largely relied upon laborious dissections, endoneurocranial tissue displacement, and physical measurement to derive comparative volumes. As an alternative, we present a new precise calculation method based upon coupled magnetic resonance (MR) imaging and computed tomography (CT). Our approach utilizes a novel interactive Fakir probe cross-referenced with an automated CT protocol to efficiently generate total volumes and surface areas of the brain tissue and endoneurocranial space, as well as the discrete cephalic compartments. We also complemented our procedures by using sodium polytungstate (SPT) as a contrast agent. This greatly enhanced CT applications but did not degrade MR quality and is therefore practical for virtual brain tissue reconstructions employing multiple imaging modalities. To demonstrate our technique, we visualized sex-based brain size differentiation in a sample set of Ring-necked pheasants (*Phasianus colchicus*). This revealed no significant variance in relative volume or surface areas of the primary brain regions. Rather, a trend towards isometric enlargement of the total brain and endoneurocranial space was evidenced in males versus females, thus advocating a non-differential sexually dimorphic pattern of brain size increase amongst these facultatively flying birds.

Cognitive, behavioural, ecological and sensory-motor mechanisms of the central nervous system (CNS) in vertebrates have been inferred from total size and dimensional compartmentalization of the brain and endoneurocranial space; however, the manual procedures for measuring these structures are known to be imprecise^1^. This has critical implications for correlative neuroanatomy as well as the reconstruction of fossils in which the neural tissues do not preserve. Moreover, detailed mapping of intra/interspecific variability^2^ and developmental trajectories^3^ is vital because these can influence the interpretation of evolutionary modifications in the CNS through time. Advanced digital imaging technologies offer an optimal tool for accurate quantitative investigations of complex neural structures. Yet, the technical manipulation of virtual tomographic data for specialized evolutionary studies is still a nascent field^4^,^5^,^6^,^7^. This contribution therefore presents a new benchmark protocol for 3D visualization and rigorous volumetric measurement of the brain and endoneurocranial space in vertebrates. The utility of our technique is demonstrated via a case study of differential sex-related size modularization of the main cerebral divisions in an extant representative of Phasianidae (Galliformes). This particular problem is of interest because overall brain enlargement is thought to result from relative dimensional increases in major components according to their importance^8^. Nevertheless correlation between behavioural complexity and size of the brain is not entirely clear^1^. Modular changes are also potentially independent within each neurological region^9^, but have not yet been tomographically compared against sexual dimorphism, which appears to select for larger total brain volumes in the males of some species^10^,^11^,^12^,^13^. Furthermore, our experimental model of the Ring-necked pheasant (*Phasianus colchicus*), a mid-sized (<one meter maximum snout-tail length and up to ~3 kg) facultative terrestrial bird that is capable of short-distance flight, represents a viable proxy for the lifestyle and locomotive capabilities of early stem avians^14^,^15^,^16^. These likewise trended towards gross brain enlargement via differential development of key cerebral components^17^,^18^,^19^,^20^.

Our analysis methods employed a combination of Magnetic resonance (MR) imaging, a gold standard for intact brain visualization, and Computed tomography (CT) as a superior modality for digitally recreating the endoneurocranial space. These were augmented by innovative interactive software coupled with automated segmentation procedures to enable fast and efficient compilation of accurate cross-referenced volumetric measurements. Experimentation with different soft tissue fixation parameters and the inclusion of sodium polytungstate (SPT) as a contrast agent^21^ also illustrates an ideal sample treatment technique that can be used for discriminating detailed surface geometry and spatial relations between the brain tissue and endoneurocranium, as well as internal segregation of major brain divisions (prosencephalon, mesencephalon and rhombencephalon) in an avian paradigm of other vertebrates.

## Results and discussion

**Figure 1:**
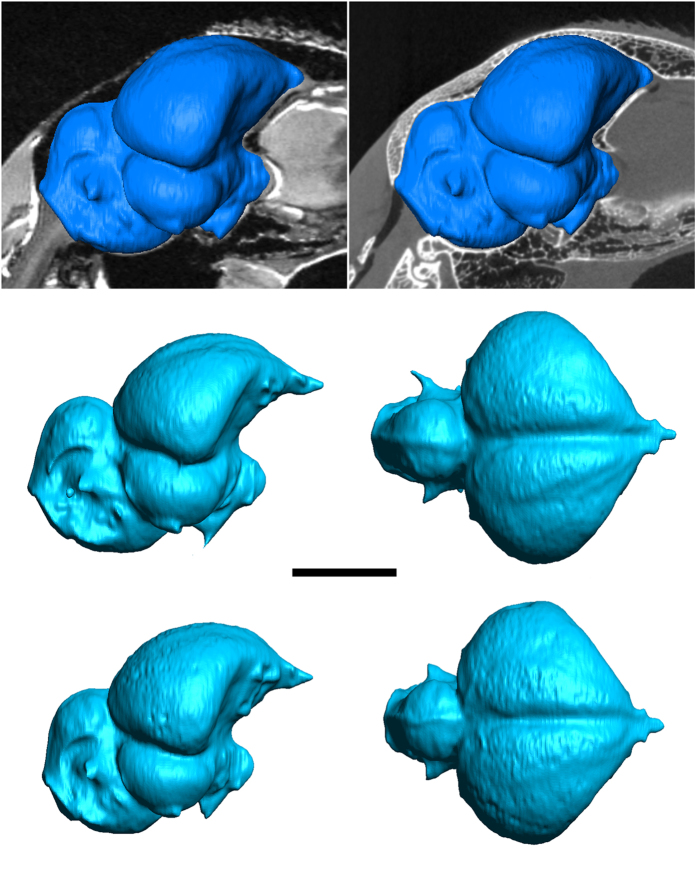
Automated surface renderings of segmented avian endoneurocrania (*Phasianus colchicus*). Mid-sagittal section of (**a**) 3D RARE MR versus Watershed method CT (**b**) generated image with inset endoneurocranial surface (adult male specimen). Comparative proportions of male (**c**,**d**) and female (**e**,**f**) endoneurocrania in lateral (**c**,**e**) and dorsal (**d**,**f**) views.

**Figure 2:**
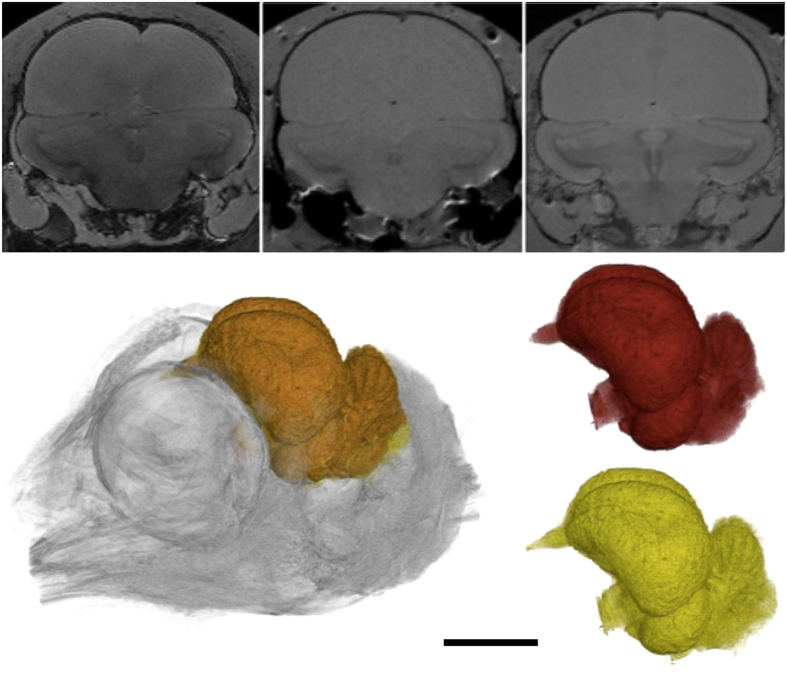
Effects of SPT fixation on avian brain tissue (*Phasianus colchicus*). MR visualization of juvenile specimen after: (**a**) three days with no SPT; (**b**) six days with SPT + water; (**c**) three months with SPT + formalin. Surface rendering of 3D RARE MR data (adult male specimen) illustrating: (**d**) the *in situ* endoneurocranial space; (**e**) isolated brain tissues; (**f**) and isolated endoneurocranial space.

Our MR and μCT data acquisitions obtained sufficient contrast and resolution, as well as a high signal-to-noise ratio necessary for quantitative analysis. Image artefacts and bulk motion from scanner vibration were completely negated. The addition of SPT substantially enhanced CT visualization (Fig. 1a,b; Supplementary Figure 1). Moreover, it did not degrade MR image quality; on the contrary, some brain structures possessed better contrast (Fig. 2a–c) after prolonged fixation, and were measurable through a marked increase in the signal-to-noise ratio after three months (83.5+/−0.8) versus six days (51.5+/−0.9) of immersion. Screening for MR contrast deterioration during prolonged scanning (Supplementary Figure 2) also revealed <1% standard deviation between normalized images (T_2_ relaxation time = 64.6+/−2.0 ms at commencement as opposed to 63.8+/−3.2 ms after 16 hours). Relaxometry, on the other hand, returned only marginal improvement: T_1_ = 2170.5+/−3.5 ms (4% increases), T_2_ = 1606.8+/−0.6 (10% decreases). These results compliment recent findings by Kotrotsou *et al.*^22^, who demonstrated that post-mortem subject volume variation was significantly reduced across time points relative to inter-subject volume variation over one week/six months. Kotrotsou *et al.*^22^ further concluded that *ex vivo* and *in vivo* brain volume metrics are linearly correlated, thus *ex vivo* MR volumetry can accurately capture ante-mortem endoneurocranial anatomy. The most important outcome of this study, however, was that our combined technique of incorporating an interactive seven-fold Fakir probe for MR with an automated Watershed application for CT (both detailed in the materials and methods section) produced complementary information about brain versus interstitium/endoneurocranial volume and surface area (Table 1). Our endoneurocranial values differed by 8.0 ± 1.3%, probably reflecting manual segmentation error from poorer MR imaging of bone^23^. In contrast, MR measurements of brain soft tissues were found to deviate by only 0.4+/−1.9% (Table 1, Supplementary Figure 3).

The benefits of using MR to visualize brain structures are well known^6^,^24^, and have been illustrated elsewhere in extant avians^23^,^25^,^26^. However, indistinct discrimination of the osseous interstitial border is usually overcome by CT co-registration of both modalities with automated segmentation^24^. Our new procedure of separate computation for each major brain compartment (Supplementary Figure 4) augments this approach by contributing robustly correlated volume fractions and surface areas (Table 2 and 3; Pearson’s correlation coefficient of volume versus surfaced weighted mean distance = 0.997). Such accuracy demonstrates that reliable data can be derived from our rapid processing procedure, and that a combination of MR and CT offers a very precise means of generating cross-referenced volumetric calculations of not only proportional values but also total brain space parameters (including *in* (including *in vivo*^22^) across the entire endoneurocranium.

Critical to our particular test case was the discovery that no marked differentiation existed in compartmental measurements between the sexes of our adult-stage *Phasianus colchicus* sample (Table 2 and 3). Conversely, distinct variation (p < 0.05) occurred in absolute volume/surface area with males being demonstrably larger than 10% (Fig. 1c–f; Table 1). Although it is clear that fundamental changes in the separate brain parts do alter overall brain size, our precise calculations detected an isometric, perhaps hormonally driven^1^ increase that accords with other documented examples of avian sexual dimorphism^10^,^11^,^12^,^13^; a phenomenon similarly influencing gross brain size in mammals^27^,^28^. This effect is presumably coupled with other intraspecific proportional changes including ontogenetic variability^3^ and ecomorphology (e.g. lifestyle specific and seasonal fluctuations^1^,^2^) to overlay the intrinsic mosaic evolution of avian^9^ and mammalian^29^ brain development. As an extension, dimorphic segregation might also be discernible amongst the restored brains of fossil avians, and could be used to discriminate disputed sexual morphs accepting the constraints of adequate diagenetic retro-deformation and sample size^30^,^31^,^32^,^33^. More importantly, exact quantitative measure of different brain regions, especially the proportional enlargement of the forebrain, which has been qualitatively contested as an indicator of enhanced somatosensory integration and the capacity for flight^19^,^20^, requires further investigation. Likewise, relative surface areas of key endoneurocranial compartments particularly those housing the avian eminentia sagittalis^34^ (=exterior expression of the upper hyperstriatum or Wulst^35^) would greatly assist with reconstructing comparative visual evolution and palaeoecology relative to extant analogues^36^. Given this substantial potential, the broader application of optimizing the efficiency of existing MR and CT analyses offers a significant advancement for both comparative neurology and virtual tissue visualization in biological research.

## Methods

### Sample preparation and scanning

All animal experimentation protocols were approved by the Ethics Committee of the Institute for Clinical and Experimental Medicine and the Ministry of Health of Czech Republic in accordance with the European Communities Council Directive 86/609/EEC. The severed heads of six adult (six months old; three male, three female), and four early stage juvenile (seven weeks old; two male, two female) *Phasianus colchicus* were fixed in formalin for four weeks. These specimens were each sealed in airtight plastic bags to prevent drying and shrinkage^1^. They were then scanned at high resolution (voxel volume = 0.002775 mm^3^) using a 4.7T MR spectrometer (Bruker BioSpec) equipped with a commercially available resonator coil (Bruker), and 3D Rapid Acquisition incorporating a Relaxation Enhancement (RARE) multi-spin echo sequence (basic parameters in Table 4). Prolonged MR scan time (>13 hours) generated images with sufficient contrast-to-noise ratio for quantitative analysis (Supplementary Figure 3c, 4, 5). We also processed one adult control sample of a Hoatzin (*Opisthocomus hoazin*) using identical procedures to examine possible changes in MR contrast using 2D RARE multi-spin echo sequence (Table 4). Hampering of image contrast during long term MR scanning was assessed via relaxometry undertaken at 25 °C with a standard Car-Purcel-Meiboom-Gill (CPMG) multi-spin echo sequence. Two T_2_ maps (Table 1) were calculated with an evolution delay of 17 hours between maps. Each sample was retained within the MR scanner for the full duration of the study (>20 hours). CT data was acquired on an *Albira* (Bruker BioSpin) μCT/PET system scanner and reconstructed using the Feldkamp, Davis, and Kress (FDK) algorithm in *Plastimatch*^37^ (resolution = 125 × 125 × 125 μm^3^).

To directly compare our MR and μCT results, each sample was immersed in a saline solution mixed with neutral formalin and SPT to enhance CT contrast. The effects of SPT were also examined relative to early-stage *ex vivo P. colchicus* ontogenetic proxies scanned under varying conditions (contrasting tissue preparation and media): (1) saline solution + neutral formalin but without SPT after 3 days of fixation; (2) an SPT water solution (50g/l) after 6 days of fixation; and (3) a saline + neutral formalin + SPT solution after 3 months of fixation. Because relaxation times determine contrast of MR images, we additionally measured T_1_ (saturation recovery sequence) and T_2_ (CPMG sequence) of fixative medium with and without SPT on a 0.5T relaxometer (Bruker Minispec) at 25 *°*C (Supplementary Table 1). Our MR images differed according to the dominant influence (weighting) of proton density during T_1_ or T_2_ (T_2_*) relaxation times. This was due to chemical fixation of the tissue by formalin (identified via comparisons with *in vivo* samples), which encourages protein cross-linking and immobilization of water molecules, and thus a decrease in T_2_ relaxation times^38^,^39^. Fixation effects are dependent on agent concentrations, scan time, and positioning within the sample^39^,^40^,^41^,^42^. Dawe *et al.* showed that T_2_ values near the surface of human brain hemispheres are constant during six-month fixation periods, and therefore longer fixation times (>three months) are necessary to stabilize T_2_ relaxation times throughout the whole tissue body^42^. To accommodate, we used concentrated media and proportionately small avian brains to achieve complete fixation in about four weeks; evidenced by stable T_2_ values and MR contrast.

### Volumetric calculations

Brain tissue and endoneurocranial volumes/surface areas were calculated from manual delineation in *VGStudio MAX* (http://www.volumegraphics.com), and employed as a comparison for Fakir method and Watershed segmentation protocols. We also developed a new software package based on the Fakir method for interactive manipulation of our MR data. The Fakir method estimates the volume and surface area of visualized objects from their intersections with a random 3D grid of lines (Supplementary Figure 5). Cruz-Orive^43^ originally developed the Fakir probe with a single set of parallel lines, but this suffered from estimation variance because the line intersection lengths differed directionally, thus impinging measurement of anisotropic objects. Sandau^44^ included perpendicular fixation of the grid with an orthogonal triplet of parallel lines, yet again this required isotropic orientation of surface elements for non-biased surface area estimation. The optimized Fakir method^45^ has solved these problems by employing multiple grids with a random orientation. These benefit from an antithetic effect (negative covariance of partial estimates from multidirectional lines^46^) to decrease variance. As an example, the maximum coefficient of variation (CV) achieved for planar objects is 0.58 for a single probe, but can be significantly improved via a triple probe (CV = 0.10). To elaborate this further, we initiated a seven-fold probe^47^, which achieved an orientation CV < 0.04. Grid geometry was further enhanced by mutually adjusting the sets of parallel lines to halve the number of intersects across the given surface area required for achieving precision. More information is available from: http://www2.biomed.cas.cz/~janacek/fakir/3dtools.htm.

To increase speed and efficiency, we also used an automated 3D image analysis for μCT. This utilized a Watershed method, which is a powerful tool based on basin-like contour forms defined by closed peaks and troughs^48^,^49^. Such a technique offers marked advantage for image segmentation, and is augmented by fast computation times in comparison to other approaches. We implemented our Watershed algorithm in purpose-developed modules using *Amira* (FEI).

## Additional Information

**How to cite this article**: Jirak, D., Janacek, J. & Kear, B. P. A combined MR and CT study for precise quantitative analysis of the avian brain. *Sci. Rep.* **5**, 16002; doi: 10.1038/srep16002 (2015).

## Supplementary Material

Supplementary Information

## Figures and Tables

**Table 1:** Volumetric and surface area calculations for total brain tissue versus endocranial space of both adult female and male *Phasianus colchicus.*

Segmentation	Total Brain Tissue		Total Endocranium	
	Female	Male	Female	Male
MR manual volume (mm^3^)	3335+/−84	3752+/−154	4003+/−103	4344+/−160
Volume (mm^3^)	•3383+/−131	•3729+/−197	••3642+/−52	••4036+/−172
Surface area (mm^2^)	•1394+/−39	•1538+/−80	••1500+/−37	••1572+/−91

^•^MR and Fakir.

^••^CT and Watershed methods.

**Table 2:** Brain compartment volumes (mm^3^) and surface areas (mm^2^).

Specimen	Prosencephalon		Mesencephalon		Rhombencephalon		Total	Total
	Volume	Area	Volume	Area	Volume	Area	Volume	Area
Male 1	2363	922	652	277	726	301	3741	1500
Male 2	2229	882	563	296	734	306	3526	1485
Male 3	2375	890	661	370	883	370	3919	1630
Female 1	2343	893	511	248	638	267	3493	1408
Female 2	2204	882	570	262	643	280	3418	1424
Female 3	2026	808	553	256	660	285	3238	1350
Juvenile male 1	1784	758	443	222	558	293	2785	1273
Juvenile male 2	1964	734	492	235	612	309	3068	1278
Juvenile female 1	1913	829	437	222	569	312	2919	1363
Juvenile female 2	1731	692	425	262	579	351	2735	1305

Calculated using the enhanced Fakir probe on MR data.

**Table 3:** Brain compartments volumes (mm^3^) and surface areas (mm^2^) relative to equivalent total brain measurements.

Specimen	Prosencephalon		Mesencephalon		Rhombencephalon	
	Volume	Area	Volume	Area	Volume	Area
Male 1	0.63	0.61	0.17	0.18	0.19	0.20
Male 2	0.63	0.59	0.16	0.20	0.21	0.21
Male 3	0.61	0.55	0.17	0.23	0.23	0.23
Female 1	0.67	0.63	0.15	0.18	0.18	0.19
Female 2	0.65	0.62	0.17	0.18	0.19	0.20
Female 3	0.63	0.60	0.17	0.19	0.20	0.21
**Mean (adult)**	**0.64 ± 0.02**	**0.60 ± 0.03**	**0.16 ± 0.01**	**0.19 ± 0.02**	**0.20 ± 0.02**	**0.21 ± 0.01**
Juvenile male 1	0,64	0,60	0,16	0,17	0,20	0,23
Juvenile male 2	0,64	0,57	0,16	0,18	0,20	0,24
Juvenile female 1	0,66	0,61	0,15	0,16	0,19	0,23
Juvenile female 2	0,63	0,53	0,16	0,20	0,21	0,27
**Mean (juvenile)**	**0.64 ± 0.01**	**0.58 ± 0.04**	**0.16 ± 0.01**	**0.18 ± 0.02**	**0.20 ± 0.01**	**0.24 ± 0.02**

Calculated using the enhanced Fakir probe on MR data.

**Table 4:** Parameters for RARE and relaxometry sequences.

Parameters	3D RARE	2D RARE	Relaxometry
Repetition time (*T*_R_ ms)	500	3000	5000
Effective echo time (*T*_E_ ms)	36	36	7.2
Turbo factor	8	8	−
Number of acquisitions	8	2	8
Field of view (FOV cm^3^)	5.0 × 6.2 × 6.0	4.6 × 4.6	4.6 × 4.6
Spatial resolution (μm^2^)	98 × 121	180 × 180	359 × 359
Effective spectral bandwidth (Hz)	69444	34722	101010
Scan time (hrs)	18.12	2.24	1.25
Slice thickness (mm)	0.234	2	2
Echo number	8	8	256
