# Supplementary material for: A combined MR and CT study for precise quantitative analysis of the avian brain
Source: Sci Rep. 2015 Oct 30;5:16002. doi: 10.1038/srep16002 (PMC4626839; doi:10.1038/srep16002)
Supplement: Supplementary Information [file srep16002-s1.pdf]

Supplement for:

A combined MR and CT study for precise quantitative analysis of the avian brain.

Daniel Jirak[1,2], Jiri Janacek[3] & Benjamin P. Kear[4,5]

[1]MR Unit, Department of Diagnostic and Interventional Radiology, Institute for Clinical and Experimental Medicine, Vídeňská 1958/9 140 21 Prague, Czech Republic, [2]Institute of Biophysics and Informatics, 1st Medicine Faculty, Charles University, Prague, Czech Republic, [3]Department of Biomathematics, Institute of Physiology, The Czech Academy of Sciences, Vídeňská 1083 142 20 Prague, Czech Republic, [4]Museum of Evolution, Uppsala University, Norbyvägen 16, SE-752 36 Uppsala, Sweden. [5]Palaeobiology Programme, Department of Earth Sciences, Uppsala University, Villavägen 16, SE-752 36 Uppsala, Sweden.

Correspondence and requests for material should be sent to: Daniel Jirak email: daji@medicon.cz

Additional information for Results and Discussion

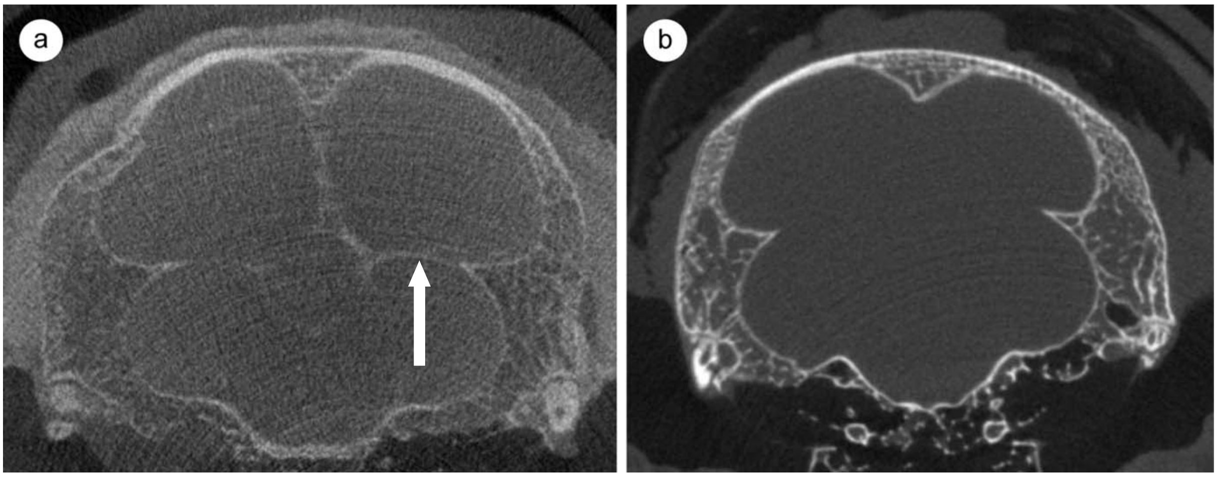

Supplementary Figure 1 | Comparative effects of SPT treatment for improved CT contrast of intracranial structures in adult *Phasianus colchicus*. (a) Visualization of a male specimen with SPT showing enhanced contrast between major brain tissue regions; (b) female specimen with no internal structures detected when SPT was withheld. Arrow indicates internal structures visible only after SPT treatment.

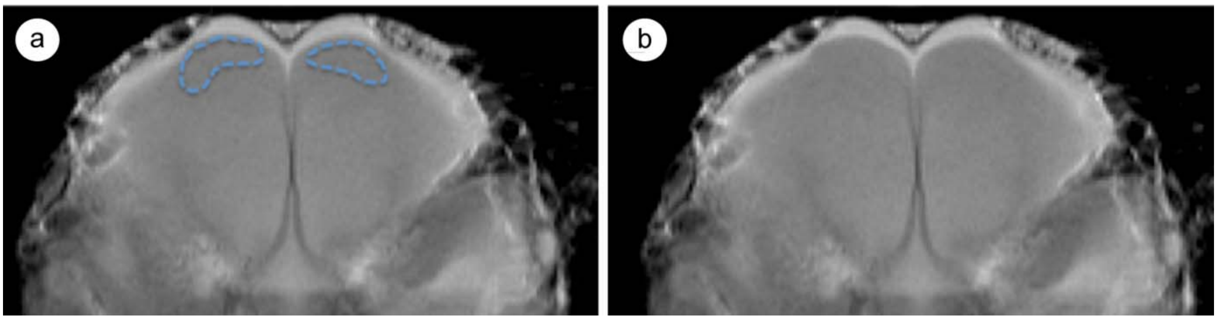

Supplementary Figure 2 | MR imaging (2D RARE) of a control sample (*Opisthocomus hoazin*) to test the effects of prolonged scan time. (a) Visualization immediately following removal from medium; (b) visualization after 13 hours prolonged scanning. Highlighted area (dashed line) was targeted for comparative T_2 calculations.

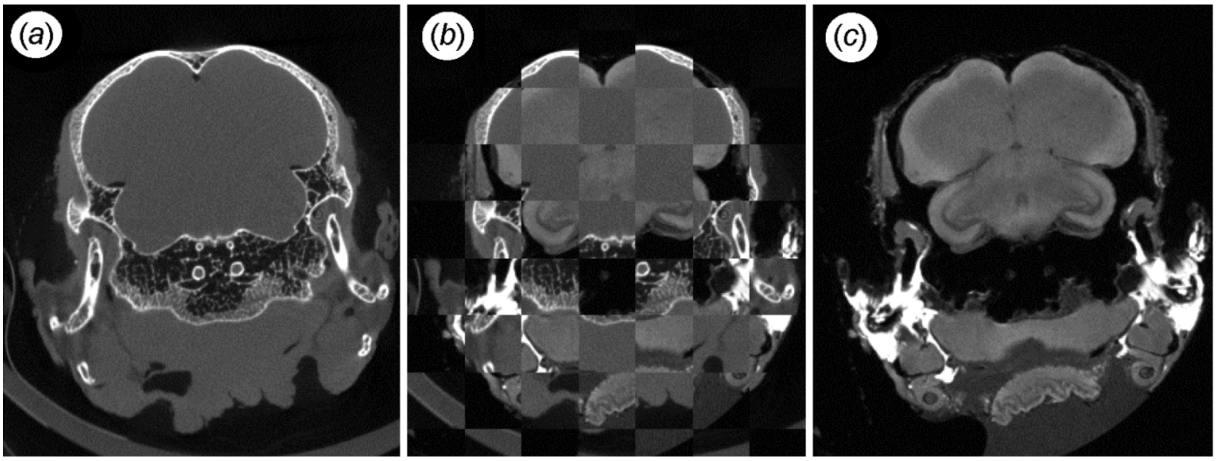

Supplementary Figure 3 | Co-registration of CT and 3D RARE MR images of the cranium from an adult (male) ***Phasianus colchicus***. (a) CT image; (b) checkerboard mosaic image (from CT and MR) registered by mutual information minimization in 3D; (c) MR image.

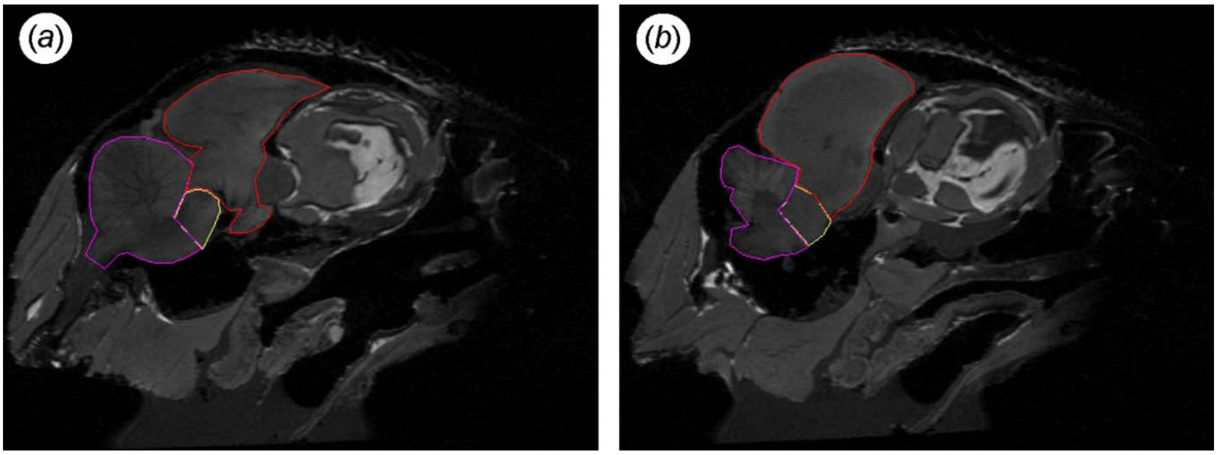

Supplementary Figure 4 | Delineation of brain compartments in 3D RARE MR images of the cranium from an adult (male) ***Phasianus colchicus*.** (a) Saggital middle plain; (b) saggital plane 2.7 mm from the middle plain. Colour demarcation: red = prosencephalon; yellow = mesencephalon; purple = cerebellum and hindbrain (rhombencephalon). Compartmental areas were delineated manually based on Karten *et al*.[1]

References

Karten, H. J., Brzozowska-Prechtl, A., Lovell, P. V., Tang, D. D., Mello, C. V., Wang, H., & Mitra, P. P. Digital atlas of the zebra finch (*Taeniopygia guttata*) brain: A high-resolution photo atlas. *J. Comp. Neurol.* **521**, 3702–3715 (2013).

Additional information for Methods

Supplementary Table 1 | Parameters for relaxometry measurements of T_1/T_2 versus SPT. Abbreviations: •T_1, utilizes saturation recovery sequence; ••T_2, utilizes CPMG sequence.

Parameters	•T_1	••T_2
Recycle delay (s)	10	5
Number of points	10	2000
Duration factor	1.85	-
Pulse separation (ms)	-	1
Echo number	-	2000

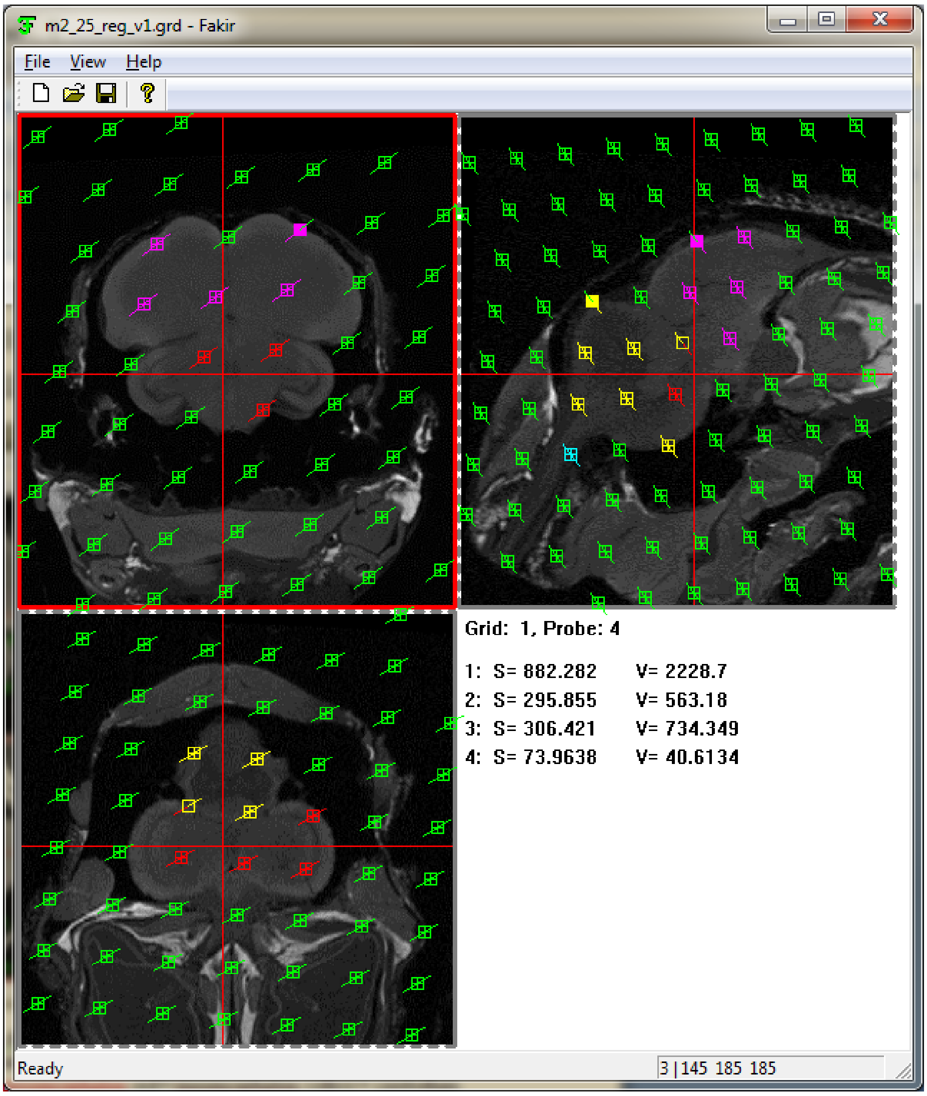

Supplementary Figure 5 | Screen capture of the Fakir probe compartmental brain volume measurements from an adult (male) *Phasianus colchicus*. Orthoslice includes one of seven parallel line sets. Colour demarcation: green = non-brain tissues or space; purple = prosencephalon; red = mesencephalon; yellow = cerebellum and hindbrain (rhombencephalon); cyan = spinal cord. Filled squares delimit the brain surface.
